# Melatonin enhances sorafenib-induced cytotoxicity in FLT3-ITD acute myeloid leukemia cells by redox modification: Erratum

**DOI:** 10.7150/thno.118171

**Published:** 2025-06-15

**Authors:** Tian Tian, Jiajun Li, Yizhuo Li, Yun-Xin Lu, Yan-Lai Tang, Hua Wang, Fufu Zheng, Dingbo Shi, Qian Long, Miao Chen, Guillermo Garcia-Manero, Yumin Hu, Lijun Qin, Wuguo Deng

**Affiliations:** 1 Sun Yat-sen University Cancer Center; State Key Laboratory of Oncology in South China; Collaborative Innovation Center for Cancer Medicine, Guangzhou 510060, China; 2 Sun Yat-sen Memorial Hospital, Sun Yat-sen University, Guangzhou 510000, China.; 3 The First Affiliated Hospital, Sun Yat-sen University, Guangzhou 510080, China; 4 Department of Leukemia, The University of Texas MD Anderson Cancer Center, Houston, Texas 77030, USA.

The authors regret some incorrect flow cytometry scatter diagrams were accidentally displayed during data organization in Figure 1C, Figure 2F and Figure 3F. The authors confirm that these corrections do not change the result interpretation or conclusions of the article. The authors apologize for any inconvenience that the errors may have caused.

## Figures and Tables

**Figure 1:**
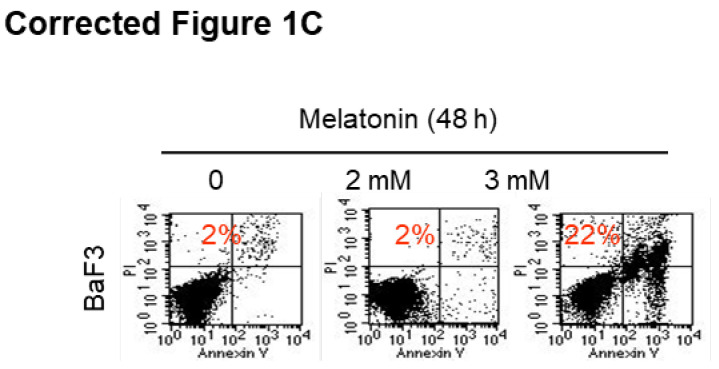
** (C)** Representative images of cell apoptosis in the indicated cells treated with melatonin for 48 h were determined by Annexin-V/propidium iodide (PI) assay (red numbers indicate subpopulation of cells negative for Annexin V/PI).

**Figure 2:**
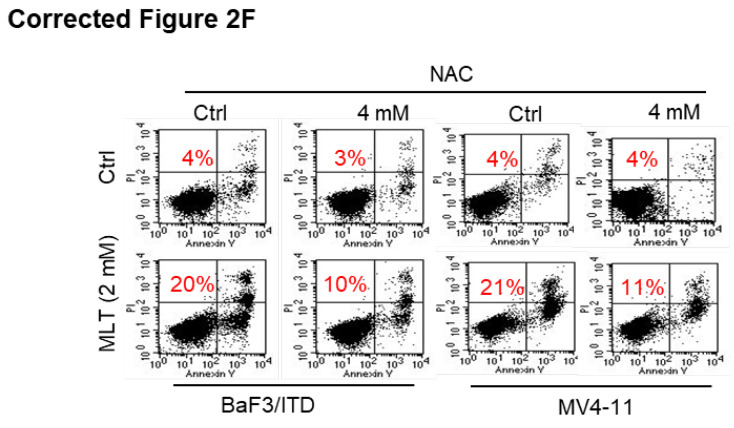
** (F)** Reversion of Melatonin-induced cell death by the antioxidant NAC (*N*-acetyl-*L*-cysteine). BaF3/ITD and MV4-11 cells were treated with melatonin (2 mM) alone or a combination NAC (4 mM) for 48 h, then determined by Annexin-V/propidium iodide (PI) assay (red numbers indicate subpopulation of cells negative for Annexin V/PI).

**Figure 3:**
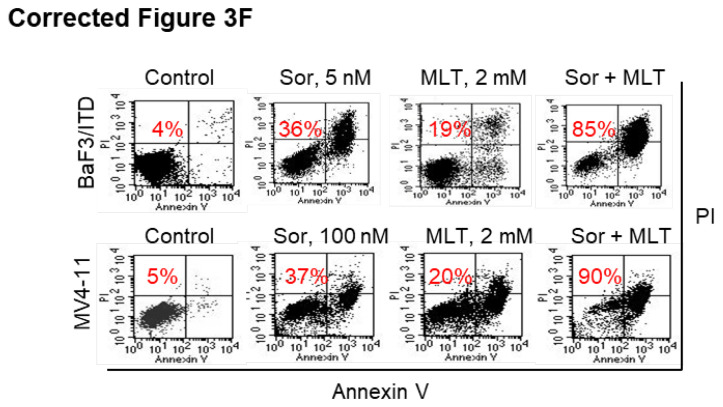
** (F)** Representative images of cell apoptosis in the indicated cells treated with melatonin, sorafenib or combination for 48 h (red numbers indicate subpopulation of cells negative for Annexin V/PI).

